# Sensitivity and Specificity of Double-Track Sign in the Detection of Transverse Sinus Stenosis: A Multicenter Retrospective Study

**DOI:** 10.1371/journal.pone.0135897

**Published:** 2015-08-20

**Authors:** De-Sheng Zhu, Jue Fu, Yi Zhang, Chong Xie, Xiao-Qing Wang, Yue Zhang, Jie Yang, Shi-Xu Li, Xiao-Bei Liu, Zhi-Wen Wan, Qiang Dong, Yang-Tai Guan

**Affiliations:** 1 Department of Neurology, Ren Ji Hospital, School of Medicine, Shanghai Jiao Tong University, Shanghai, China; 2 Department of Neurology, Huashan Hospital, Fudan University, Shanghai, China; 3 Department of Pathology, Fuzhou Medical College, Nanchang University, Fuzhou, China; 4 Department of Neurology, Changhai Hospital, Second Military Medical University, Shanghai, China; 5 Department of Neurology, Nanyuan Hospital, Beijing, China; 6 Department of Neurology, Dong Fang Hospital, School of medicine, Tong Ji University, Shanghai, China; Affiliated Hospital of North Sichuan Medical College, CHINA

## Abstract

**Background:**

Transverse sinus stenosis (TSS) is common among patients with cerebral venous sinus thrombosis. No previous studies have reported on double-track sign detected on axial Gd-enhanced T1WI in TSS. This study aimed to determine the sensitivity and specificity of the double-track sign in the detection of TSS.

**Methods:**

We retrospectively reviewed medical records of 383 patients with transverse sinus thrombosis (TST) and 30 patients with normal transverse sinus from 5 participating hospitals in china from January 2008 to June 2014. 167 feasible transverse sinuses included in this study were categorized into TSS (n = 76), transverse sinus occlusion (TSO) (n = 52) and transverse sinus normal (TSN) groups (n = 39) according to imaging diagnosis on digital subtraction angiography (DSA) or magnetic resonance venography (MRV). Double-track sign on axial Gd-enhanced T1WI was compared among the three groups. Sensitivity and specificity of double-track sign in detection of TSS were calculated, with final imaging diagnosis of TSS on DSA or MRV as the reference standard.

**Results:**

Of 383 patients with TST recruited over a 6.5-year period, 128 patients were enrolled in the study, 255 patients were excluded because of insufficient clinical data, imaging finding and delay time, and 30 matched patients with normal transverse sinus were enrolled in the control group. Therefore, double-track sign assessment was conducted in 167 available transverse sinuses of 158 patients. Of the 76 sinuses in TSS group, 51 had double-track sign. Of the other 91 sinuses in TSO and TSN groups, 3 had a false-positive double-track sign. Thus, double-track sign on axial Gd-enhanced T1WI was 67.1% (95% CI 55.3–77.2) sensitive and 96.7% (95% CI 89.9–99.1) specific for detection of TSS.

**Conclusions:**

The double-track sign on axial Gd-enhanced T1WI is highly specific and moderate sensitive for detection of TSS. Nevertheless, it could be a direct sign and might provide an early clue for TSS.

## Introduction

Transverse sinus stenosis (TSS) is a common subtype of cerebral venous sinus thrombosis (CVST) that affects the dural venous sinus, and it accounts for about 40% of all CVST [1]. TSS contributes to intracranial hypertension and leads to severe clinical symptoms [2]. Patients with headache [3], blurred vision [4], seizure, cognitive disorder and isolated intracranial hypertension syndrome are often referred to neurologists to rule out dural venous sinus and cerebral vein causes [5, 6]. Misdiagnosis of TSS is common because of the underlying risk factors and the varied clinical manifestations [7].

Though digital subtraction angiography (DSA) is considered to be the gold standard of dural sinus stenosis, the AHA/ASA 2011 Scientific Statement recommends magnetic resonance imaging (MRI) with magnetic resonance venography (MRV) as the imaging test of choice for evaluation of suspected stenosis [8]. In most institutions, MRI is still the first-line feasible imaging method in the emergency department setting, but conventional unenhanced T1 weighted image (T1WI) MRI has a low accuracy for thrombosis, and stenosis could be exaggerated on MRV because of its potential diagnostic pitfalls related with frequent blood flow-related artifacts [9].

Currently, we frequently observed the abnormal region showed a double-track pattern on gadolinium (Gd)-enhanced T1WI in patients with TSS. We firstly named it “double-track sign”, which was caused by the contrast agent in the affected dural sinus wall, presenting with two hyperintense linear tracts of contrast agent separated by the hypointense signal of non-contrast thrombus within dural sinus. The double-track sign indicated a stenosis of transverse sinus that was confirmed by MRV and DSA, thus it may be a potential direct sign in the detection of TSS.

To our knowledge, no previous studies have reported on the value of the double-track sign in the detection of TSS. Therefore, the aim of this study was to assess the sensitivity and specificity of the double-track sign on axial Gd-enhanced T1WI in the detection of TSS via a multicenter retrospective study.

## Materials and Methods

### Ethics

This study was performed according to the principles of the Declaration of Helsinki, and was approved by the Institutional Review Board of Huashan Hospital, Fudan University. All patients’ images and medical records in the study were anonymized and de-identified prior to analysis.

### Design

This was a multicenter retrospective observational case-control study. Cases were recruited across 5 participating hospitals in China from January 2008 to June 2014. We used our clinical data base of electronic medical records in hospital to search for the key words *transverse*, *venous*, *sinus*, *thrombosis*, *stenosis* and *occlusion* to identify all hospitalized patients with transverse sinus thrombosis (TST). According to the stenosis or occlusion of transverse sinuses on DSA or MRV, available sinuses were categorized into TSS and transverse sinus occlusion (TSO) groups. Control patients with normal transverse sinus matched with TST patients were selected and their available sinuses were enrolled in the transverse sinus normal (TSN) group.

Imaging finding of the double-track sign on axial Gd-enhanced T1WI was compared among the TSS, TSO and TSN groups. Sensitivity and specificity of the double-track sign in the detection of TSS were calculated, with the final imaging diagnosis of TSS on MRV or DSA as the reference standard.

### Patients

#### Diagnosis criteria

Patients were diagnosed with TSS, TSO and TSN by DSA or MRV, based on clinical symptoms and imaging presentation. Double-track sign was identified when transverse sinus showed a double-track pattern on axial Gd-enhanced T1WI.

#### Inclusion criteria

This study only included patients with the following criteria: 1) diagnosis of TSS/TSO by DSA or MRV; 2) performed with Gd-enhanced T1WI; 3) time from MRI-to-MRV or DSA ≤ 6 hours; 4) integrity of clinical data (age, sex, clinical symptom, and comorbidities at baseline); and 5) age >18 years.

#### Exclusion criteria

Patients were excluded with one or more following criteria: 1) time from MRI-to-MRV or DSA > 6 hours; 2) images were not available for review, including unclear image finding; and 3) age≤18 years.

#### Control patients

Control patients without TST were included with the following criteria: 1) matched generally with TST patients for age and gender; 2) performed with Gd-enhanced T1WI and MRV or DSA; 3) time from MRI-to-MRV or DSA ≤ 6 hours; and 4) free from any history of neurologic or psychiatric disorders, including intracranial vascular malformations. Thirty patients were randomly selected from clinical database of electronic medical records in hospital.

### MRI Parameters

MR imaging was performed on 1.5T scanners (Magnetom Vision or Symphony; Siemens, Erlangen, Germany). The following imaging parameters were used: 1) axial T1-weighted SE-sequence (TR, 450–491 ms; TE, 10–14 ms; slice thickness, 5 mm; FOV, 210 mm; matrix size, 256; gap spacing, 30%; 1 signal intensity average, 23 slices); 2) axial T2-weighted SE-sequence, (TR, 3800–4190 ms; TE, 87–97 ms; slice thickness, 5 mm; FOV, 210 mm; matrix size, 256; gap spacing, 30%; 1 signal intensity average, 23 slices); 3) axial T1-weighted Gd-DTPA enhanced sequence (TR, 518–673 ms; TE, 9.3–14 ms; slice thickness, 5 mm; FOV, 210 mm; matrix size, 256; gap spacing, 30%; 1 signal intensity average, 23 slices); 4) Gd-DTPA MRV (TR, 2.9 ms; TE, 1.1 ms; slice thickness, 1.3 mm; FOV, 210 mm; matrix size, 256; gap spacing, 10%; 1 signal intensity average, 50 slices).

### Image Interpretation

One physician at each hospital was responsible for data collection and primary imaging assessment for TSS, TSO and TSN on DSA or MRV, and the completeness of the data collection and the repeated imaging assessment was monitored by one of the authors (De-Sheng Zhu).

Each image dataset was assessed independently by two experienced neurologists, who were blinded to patient clinical data and identification information. Image interpretation was performed on a standard PACS workstation. Reading orders were randomized, and standardized evaluation forms were used. The readers evaluated the axial Gd-enhanced T1WI for the double-track sign and had to decide whether they interpreted the presence of the double-track sign as the indicative of a TSS. The diagnostic confidence regarding the TSS was rated on a 5-point scale (1, absolutely certain; 2, very certain; 3, certain; 4, not very certain; 5, uncertain).

After having evaluated all imaging data, readers collaboratively reviewed the clinical records to take the clinical and imaging information into account. They subsequently determined, in consensus, the presence of the TSS with the reference standard based on the results of MRV or DSA. Imaging data of this study have not been reported before.

### Statistical analysis

To determine the interobserver agreement regarding the presence of double-track sign, multirater values were calculated as described in the literature. The values can range from -1.0 to 1.0, with -1.0 indicating perfect disagreement below chance, 0.0 indicating agreement equal to chance, and 1.0 indicating perfect agreement above chance [10].

Categorical variables were presented as counts and percentages and continuous variable was reported as means and standard deviation (normal distribution). Sensitivity and specificity parameters of the double-track sign for the detection of TSS, as well as their respective 95% confidence intervals (CI), were calculated. Data was analyzed using Statistical Package of the Social Sciences Software version 16.0 (SPSS, Chicago, IL, USA), setting the level of significance at a two-tailed *p*-value of <0.05.

## Results

**Fig 1:**
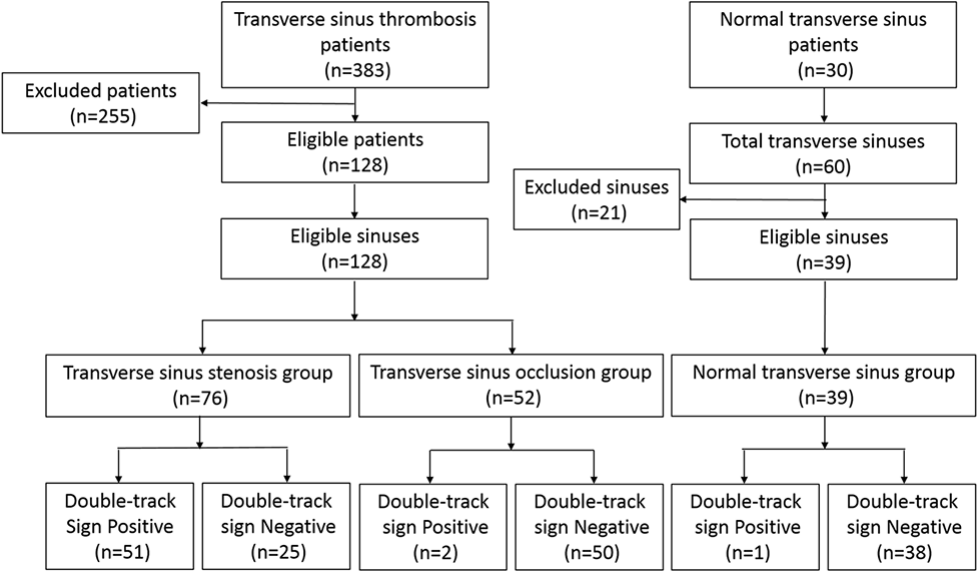
Recruitment flow diagram. Flow diagram demonstrates patient’s recruitment and imaging finding of the double-track sign.

In this study, 383 patients diagnosed with TST on DSA or MRV were recruited over a 6.5-year period (Fig 1). Of the 383 patients, 255 patients were excluded because of inconclusive clinical data, insufficient imaging finding, age factors, and delay time from MRI to MRV or DSA. Thus, 128 patients that matched the inclusion criteria were subsequently evaluated. The mean age of the 128 patients, 61% of whom were female, was 41.3 ± 14.2 years [range, 19–85 years]. Of the 128 patients with TST, none of patients was involved with bilateral transverse sinus stenosis, thus 128 transverse sinuses were included. Of the 60 transverse sinuses in the 30 control patients, 21 sinuses were excluded because of unclear image finding, and 39 available normal sinuses were included. Therefore, a total of 167 transverse sinuses were enrolled for sensitivity and specificity analysis.

**Fig 2:**
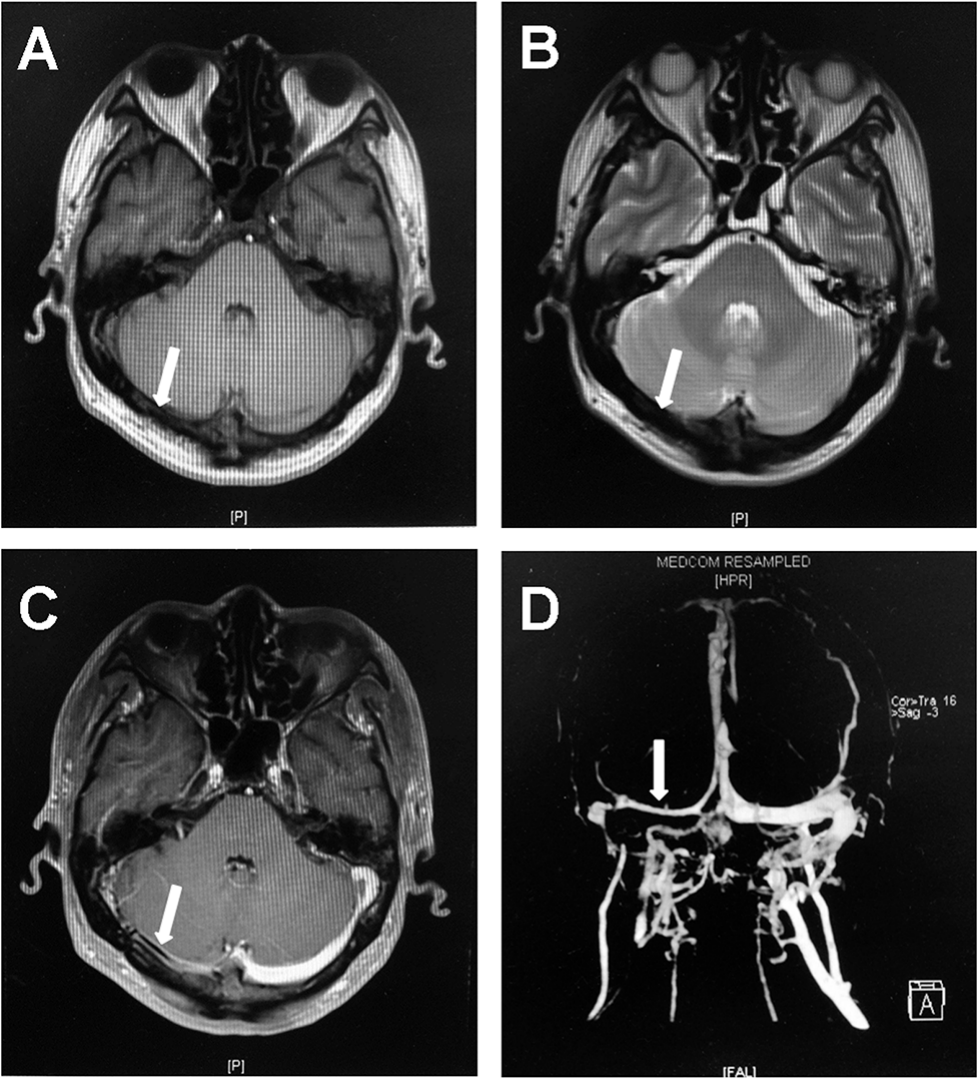
Transverse sinus stenosis with double-track sign. A 37-year old female patient with right transverse sinus stenosis. Hypointense on T1WI (A) and T2WI (B) demonstrated a chronic stage of thrombus, and double-track sign (arrow) was detected on axial Gd-enhanced T1WI (C). Transverse sinus stenosis was confirmed on MRV which showed a linear signal of blood flow in the right transverse sinus (D). (Time from MRI to MRV was 4.5 hours).

**Fig 3:**
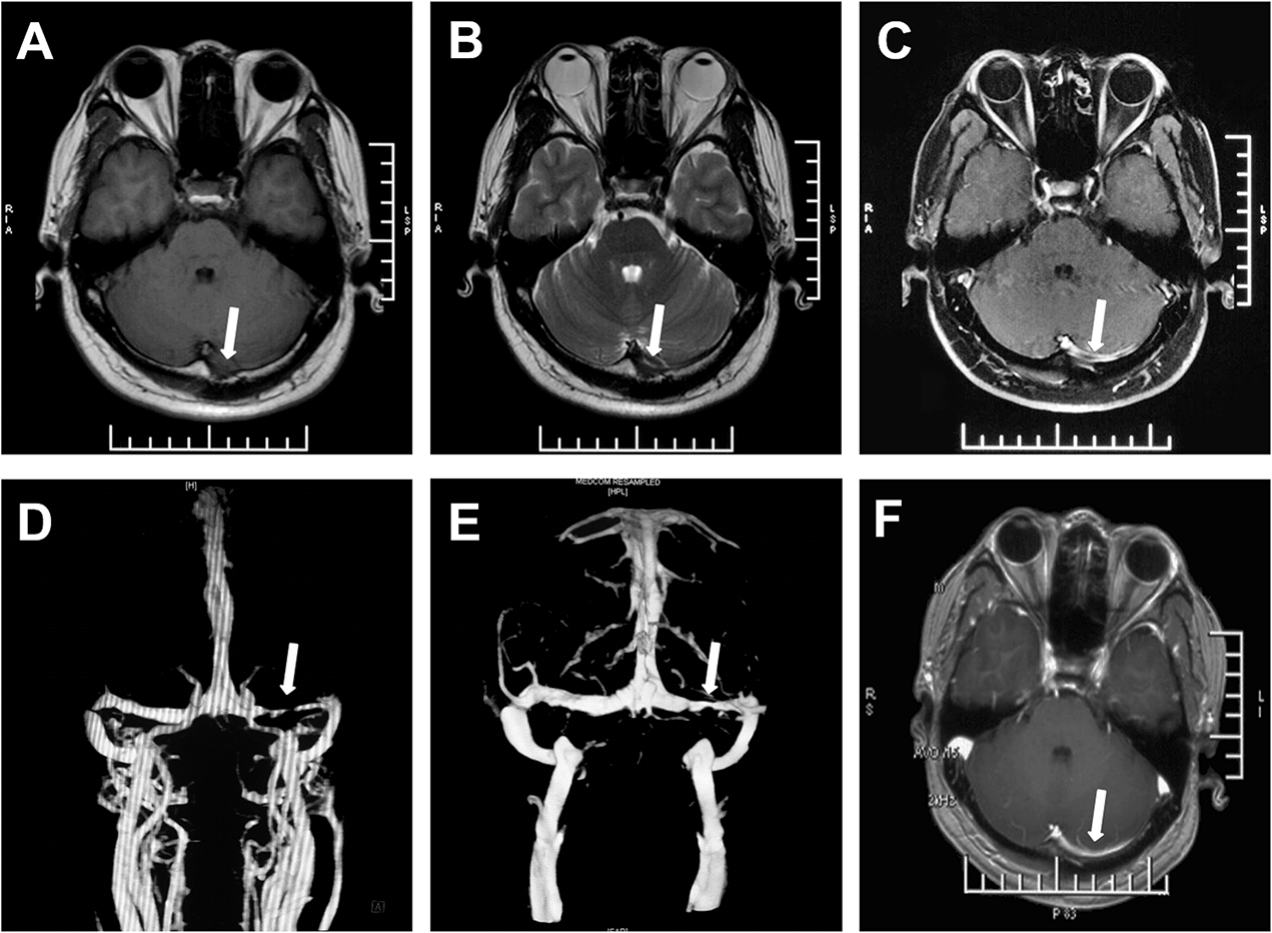
Double-track sign and sinus recanalization. A 42-year old male patient with left transverse sinus stenosis. Isointense on T1WI (A) and hypointense on T2WI (B) demonstrated an acute stage of thrombus. Double-track sign (arrow) was showed on axial Gd-enhanced T1WI (C). Transverse sinus stenosis was confirmed on MRV which showed a linear signal of blood flow in the left transverse sinus on MRV (D) (Time from MRI to MRV was 3 hours). Follow-up scan after 2 weeks of anticoagulation therapy showed that the stenosis was ameliorated than before on MRV (E), and the double-track sign disappeared on axial Gd-enhanced T1WI after the recanalization (F) (Time from MRI to MRV was 6 hours).

**Fig 4:**
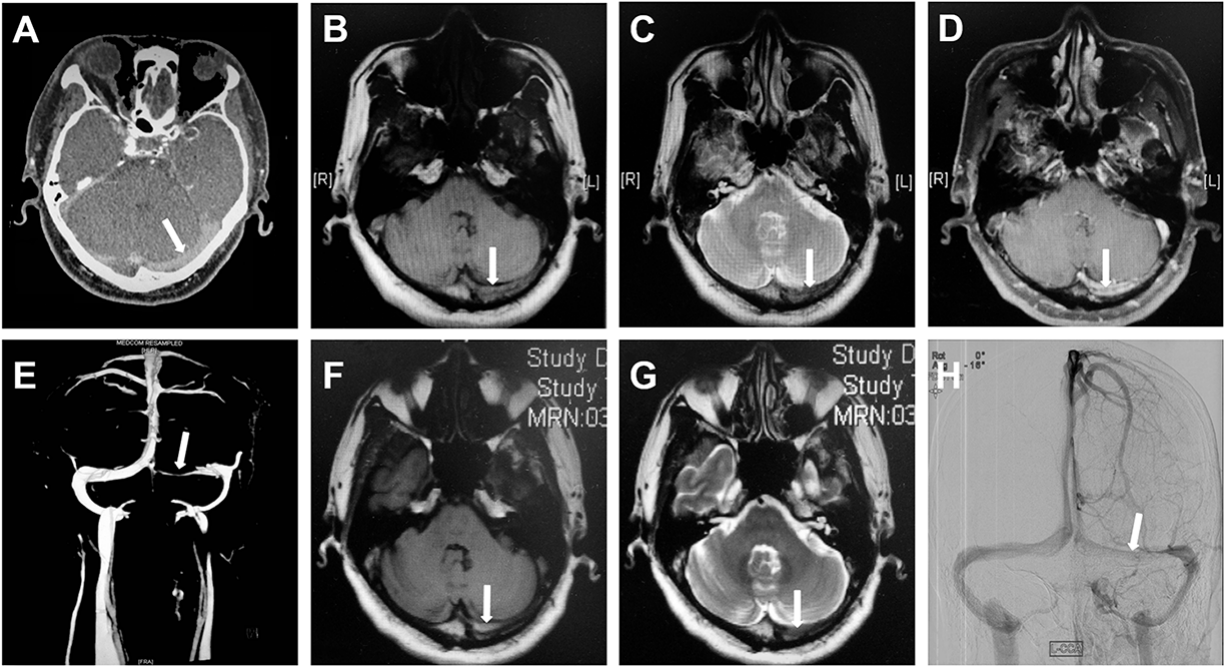
Double-track sign and the varied signal of thrombus. A 46-year old female patient with left transverse sinus stenosis. Hyperdensity of thrombus (cord sign) on non-contrast CT (A). Thrombus was isointense on T1WI (B) and hypointense on T2WI (C) which demonstrated an acute stage of thrombus. Double-track sign (arrow) was detected on axial Gd-enhanced T1WI (D). Severe stenosis was confirmed on MRV which showed a linear signal of blood flow in the left transverse sinus (E) (Time from MRI to MRV was 2.5 hours). No remission in headache in the patient after anticoagulant therapy for one week, and follow-up scan showed that the thrombus was hyperintense on T1WI (F) and hypointense on T2WI (G), which demonstrated an early sub-acute stage of thrombus. Transverse sinus stenosis was confirmed on DSA which showed the filling deficiency in the left transverse sinus (H) (Time from MRI to DSA was 3 hours).

Imaging findings of the double-track sign (Figs 2C, 3C and 4D) on axial Gd-enhanced T1WI compared to imaging diagnosis on DSA and MRV are presented in Table 1: 128 transverse sinuses of TST patients were categorized into TSS group (n = 76) and TSO group (n = 52), and 39 available normal transverse sinuses of control patients were in the TSN group (n = 39). Of the total 167 sinuses, 53 and 114 sinuses were evaluated by DSA and MRV, respectively. After identification of double-track sign on axial Gd-enhanced T1WI, double-track sign was observed in 16 and 35 sinuses evaluated by DSA and MRV, respectively, in the TSS group.

**Table 1:** Imaging findings of the double-track sign on axial Gd-enhanced T1WI compared to imaging diagnosis on DSA and MRV.

Groups	DSA		MRV		Total
	Double-track sign (+)	Double-track sign (-)	Double-track sign (+)	Double-track sign (-)	
TSS	16	7	35	18	76
TSO	0	14	2	36	52
TSN	0	16	1	22	39
Total	16	37	38	76	167

Sensitivity and specificity of the double-track sign on axial Gd-enhanced T1WI in the detection of TSS are presented in Table 2. Double-track sign assessment was conducted in a total of 167 transverse sinuses. Of the 76 sinuses in TSS group, 51 (67%) had double-track sign. Of the other 91 sinuses in TSO and TSN groups, 3 (3%) had a false-positive double-track sign. With the reference standard based on MRV, sensitivity and specificity of the double-track sign were 66.0% (95% CI 51.6–78.1) and 95.1% (95% CI 85.4–98.7), respectively, and sensitivity and specificity of the double-track sign with the reference standard based on DSA were 69.6% (95% CI 47.0–85.9) and 100% (95% CI 85.9–100), respectively. In total, the double-track sign on axial Gd-enhanced T1WI was 67.1% (95% CI 55.3–77.2) sensitive and 96.7% (95% CI 89.9–99.1) specific for the detection of TSS. The *k* values on MRV and DSA were 0.86 (95% CI 66.8–100) and 0.87 (95% CI 61.7–100), respectively, and the overall interobserver agreement for the presence of a double-track sign was 0.86 (95% CI 71.2–100).

**Table 2:** Sensitivity and specificity of the double-track sign on axial Gd-enhanced T1WI in the detection of transverse sinus stenosis.

Reference standard	Blinded Readings	Consensus Reading				Interobserver Agreement	Sensitivity	Specificity
	Double-track sign(+)	tp	fp	tn	fn	(*k* values)	(95% CI)*	(95% CI)*
MRV	38	35	3	58	18	0.86	66.0 (51.6–78.1)	95.1 (85.4–98.7)
DSA	16	16	0	30	7	0.87	69.6 (47.0–85.9)	100 (85.9–100)
Total	54	51	3	88	25	0.86	67.1 (55.3–77.2)	96.7 (89.9–99.1)

Note:—tp indicates true-positive; fp, false-positive; tn, true-negative; fn, false-negative; 95% CI, 95% confidence interval.

*Data are given in percentages.

**Fig 5:**
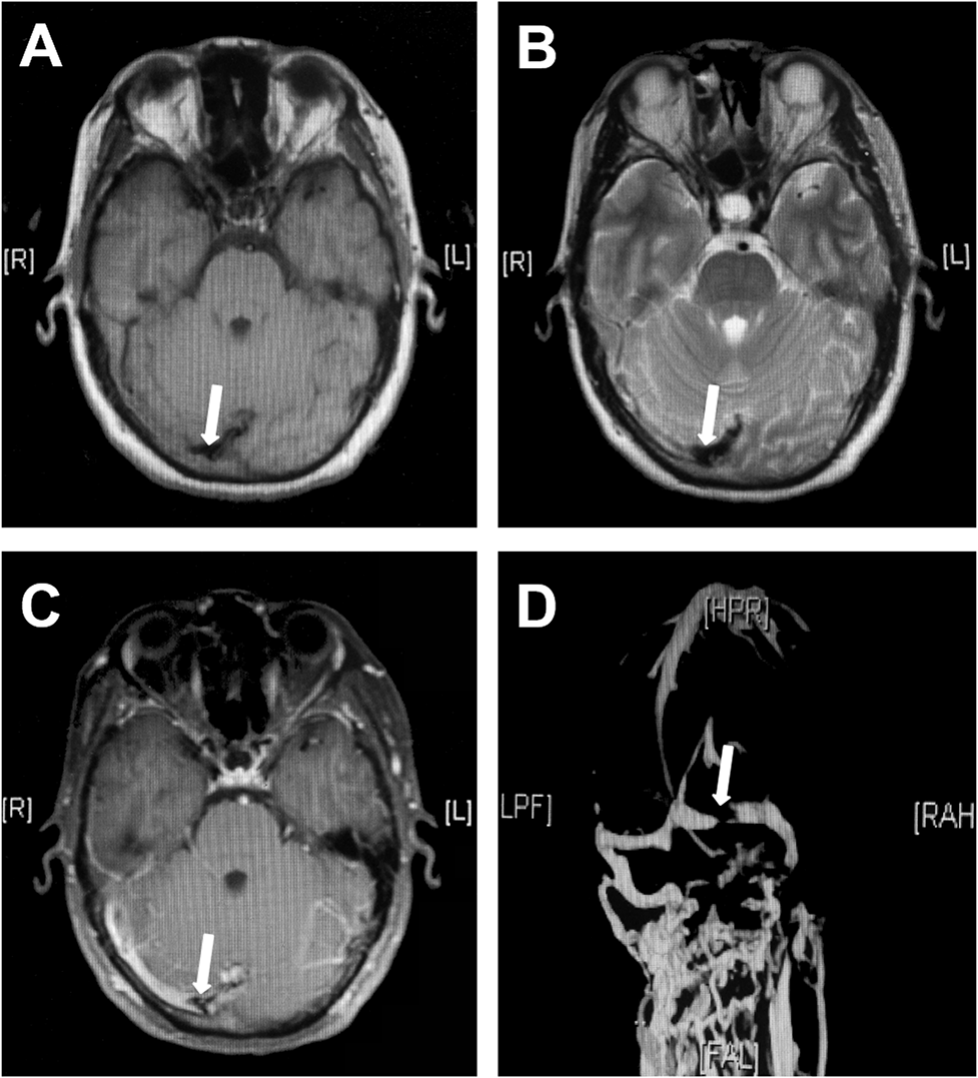
Transverse sinus occlusion without double-track sign. A 38-year old male patient with right transverse sinus occlusion. The thrombus was hypointense on T1WI (A) and T2WI (B), and double-track sign was not observed in right transverse sinus on axial Gd-enhanced T1WI (C). Transverse sinus occlusion was confirmed on MRV which showed a lack of blood flow signal in the right transverse sinus (D) (Time from MRI to MRV was 2 hours).

In the study, we observed that signal intensity of thrombus varied with the age of thrombosis within the transverse sinus on TIWI and T2WI, while the signal intensity of double-track sign was not altered on Gd-enhanced T1WI, accompanied with a linear signal of blood flow in the affected sinus on MRV or DSA (Figs 2–4). The double-track sign cannot be detected when the sinus was recanalization (Fig 3C and 3F) after anticoagulant therapy or total occlusion (Fig 5C and 5D). We also observed the cord sign on non-contrast computed tomography (CT) in some patients (Fig 4A).

## Discussion

To our knowledge, this is the first study examining the imaging finding of the double-track sign associated with TSS in cerebral vascular disease. The concept of double-track sign has been mentioned in the detection of bronchiectasis and optic neuritis on MRI and CT [11, 12], and it has been noted on sonographic examinations in pylorospasm as well [13]. In our study, the double-track sign on axial Gd-enhanced T1WI is caused by the contrast agent in the affected dural sinus wall, presenting with hyperintense signal of the venous wall without enhancement of the lumen of the traverse sinus on axial Gd-enhanced T1WI. Concerning its pathogenesis, the interaction between sinus wall cellular damage and thrombosis may play a key role in the formation of double-track sign. Regarding the anatomical structure of dural sinus, vascular endothelial cells lined on the inner surface of fibrous connective tissue within dural sinus wall, making it special intracranial venous pipelines [14–16]. Consequently, the appearance of the double-track sign may imply the jury of endothelial cells and the enhanced permeability of dural sinus wall. As thrombosis is associated with vascular, blood and hemodynamic factors [17], changes in venous wall, blood components and blood flow may develop sinus thrombosis [18, 19]. Currently, our results demonstrate that the incident ratio of TST is 1:1.6 for men and women, with a mean age at onset of 41.3 ± 14.2 years, which is consistent with other literatures [20–22].

In the present study, two linear hyperintense signals of dural sinus wall on axial Gd-enhanced T1WI, a varied signal of thrombus within transverse sinus on T1WI and T2WI, accompanied with a linear signal of blood flow within transverse sinus on MRV or DSA, were the imaging characteristics of the double-track sign. This feature provides us facilities to evaluate the TSS from sinus wall, thrombus, and blood stream simultaneously. Because CT is sensitive to fresh blood clot within dural sinus, the cord sign and delta sign on CT were observed only in one to four weeks after onset [23], and disappeared in the late sub-acute or the chronic stage of thrombosis [24]. In addition, though the unenhanced MRI T1WI is unreliable for the detection of dural venous thrombosis, MRI is more sensitive in picking up deoxyhemoglobin and methemoglobin within the thrombus, and demonstrates the age dependent signal characteristic [25]. In our study, The signal intensity of thrombus on T1WI and T2WI were varied with the age of thrombosis, on the contrary, the signal intensity of the double-track sign on axial Gd-enhanced T1WI was not altered at different stages of TSS (acute, sub-acute and chronic), and it disappeared only when a total occlusion or recanalization occurred in transverse sinus. Therefore, the double-track sign not only steadily indicated the TSS, but also illustrated further clues in prognosis of the disease. These imaging features make the double-track sign a direct sign in the identification of TSS.

Our study demonstrates that the double-track sign on axial Gd-enhanced T1WI has highly specific and moderately sensitive for detection of TSS. In this study, sensitivity and specificity of the double-track sign with the reference standard based on DSA were 69.6% (95% CI 47.0–85.9) and 100% (95% CI 85.9–100), respectively, which was higher than the sensitivity and specificity of the double-track sign with the reference standard based on MRV [66.0% (95% CI 51.6–78.1) and 95.1% (95% CI 85.4–98.7)]. This could be explained by that the accuracy of DSA in evaluating a vascular is higher than that of MRV, and a vascular stenosis may be exaggerated or misdiagnosed to be occlusion on MRV [26]. Nevertheless, the double-track sign on axial Gd-enhanced T1WI identified TSS in 51 of 76 sinuses in our study, yielding the overall sensitivity of 67.1% (95% CI 55.3–77.2) and specificity of 96.7% (95% CI 89.9–99.1). Our results demonstrate that the double-track sign is a moderate sensitivity and high specificity sign in identification of TSS. The 25 out of 76 TSS patients did not show double-track features on their Gd-enhanced T1WI, it is not related with the stage of TSS because MRI is sensitive in picking up contrast agent in sinus and deoxyhemoglobin within the thrombus, and the signal intensity of the double-track sign on axial Gd-enhanced T1WI can steadily indicate the TSS at different stage of TSS. This may be due to the fact that 2D Gd-enhanced T1WI do not provide sufficient information concerning the permeability of the venous sinus because of the frequent flow-related artifacts [27]. It is also related to the cause that sinus has soft walls which can change their shape accordingly to the intracranial pressure values and other factors [28, 29]. This pitfall is particularly important when considering the possibility of sinus stenosis because that flow-related enhancement, flow-related signal void, and slice thickness effects making T1WI extremely inaccurate [30]. Though the double-track sign cannot be proposed as a sensitive sign to detect venous sinus stenosis, it might provide an early clue for the dural sinus thrombosis. Cord sign and delta sign are considered to be classical imaging signs in detection of CVST [31]. The sensitivity and specificity of the cord sign for CVST were 64.6% and 97.2%, respectively [24]. However, cord sign on non-contrast CT has been considered to be low sensitivity and specificity in detection of CVST, because flowing venous blood generally appears to be mildly hyperattenuating, especially in children and young adults [32]. Similarly, the empty delta sign on contrast cranial CT and MRI is only presented in approximately 35% of published patients [31, 33]. Thus, the double-track sign, as well as cord sign and empty delta sign, is considered to be a unique but not sensitive sign in detection of CVST. Therefore, patients with imaging finding of double-track sign, especially those individuals combined with cord sign or delta sign, should be further evaluated by MRV or DSA to identify dural venous sinus and cerebral vein causes.

This study has some limitations. Firstly, this study was a retrospective analysis, with the nature that the study variables were not controlled or evenly distributed, and further follow-up study is needed to confirm the results. Secondly,the small sample size prevented us to drawing a solid conclusion. Only patients diagnosed of TSS/TSO by DSA or MRV and performed with Gd-enhanched MRI were included in this study, and most patients performed without Gd-enhanched MRI were excluded, which may lead to a low rate of TSS in our study and potentially decrease in the diagnostic sensitivity of the double-track sign. Thirdly, information bias existed in the course of data retrieve, and the imaging assessment varied with the timing of MRI scanning, the delay time from MRI to MRV or DSA, as well as the technical parameters. Despite the previous limitations, there are several strong points of this study. In particular, all the included patients were enrolled from wards in hospitals where they performed with a systematic medical inspection, and all information of patients were kept with electronic medical records in hospitals, which ensure the integrity and authenticity of clinical data and imaging finding. Additionally, reliable performance of imaging equipment and sophisticated technology of medical staff provided us sufficient data and clear imaging to identify the double-track sign. Finally, we rigorously managed data collection and repeated imaging assessment in our study in order to arrive at a more reliable conclusion.

In conclusion, this study demonstrates that the double-track sign on axial Gd-enhanced T1WI has highly specificity and moderate sensitive for detection of TSS. Further studies are required with larger number of patients and follow-up study to define the value of the double-track sign in the detection of TSS.
